# Human Metapneumovirus Induces Mucin 19 Which Contributes to Viral Pathogenesis

**DOI:** 10.3390/pathogens9090726

**Published:** 2020-09-03

**Authors:** Kaitlin McBride, Ma. del Rocio Banos-Lara, Nagarjuna R. Cheemarla, Antonieta Guerrero-Plata

**Affiliations:** Department of Pathobiological Sciences, Louisiana State University, Baton Rouge, LA 70808, USA; Kaitlin.elise92@gmail.com (K.M.); marocio.banos@upaep.mx (M.d.R.B.-L.); nagarjuna.cheemarla@yale.edu (N.R.C.)

**Keywords:** HMPV, Muc19, mucins, immune response, human metapneumovirus, respiratory tract, T cells, virus, lung, CD4 T cells

## Abstract

Human Metapneumovirus (HMPV) remains one of the most common viral infections causing acute respiratory tract infections, especially in young children, elderly, and immunocompromised populations. Clinical symptoms can range from mild respiratory symptoms to severe bronchiolitis and pneumonia. The production of mucus is a common feature during HMPV infection, but its contribution to HMPV-induced pathogenesis and immune response is largely unknown. Mucins are a major component of mucus and they could have an impact on how the host responds to infections. Using an in vitro system and a mouse model of infection, we identified that Mucin 19 is predominantly expressed in the respiratory tract upon HMPV infection. Moreover, the lack of Muc19 led to an improved disease, lower lung viral titers and a decrease in the number of CD4+ T cells. These data indicate that mucin 19 contributes to the activation of the immune response to HMPV and to HMPV-induced pathogenesis.

## 1. Introduction

Human metapneumovirus (HMPV) is a negative sense, single-stranded RNA virus belonging to the newly formed *Pneumoviridae* family [1]. Although HMPV was discovered in 2001, it is believed to have been present for at least 50 years [2]. HMPV infects people of all ages, but the populations most at risk for severe illness are the elderly, the immunocompromised and children [3]. In fact, by 5 years of age, virtually all children have been infected with HMPV. Symptoms of HMPV often include those common to other respiratory illnesses: rhinorrhea, cough, or fever. Some individuals with severe disease exhibit bronchiolitis or even pneumonia, and may be hospitalized [4]. Currently, there is not an effective vaccine against HMPV [5]. 

The respiratory tract has several mechanisms of protection, including the mucus layer found in its mucosal surface [6], which is increased after HMPV infection in infants [7] and the same response has been reproduced in animal models [8,9]. Mucus gels have viscous and elastic properties, many of which are credited to the physical properties and structural makeup of mucin glycoproteins, specifically gel-forming mucins. Mucins are highly glycosylated, high molecular mass macromolecules that are the major components of mucus secretions [10] and play a key role in the human airways. There are at least 15 mucins in the human lung: MUC1, MUC2, MUC3, MUC4, MUC5AC, MUC5B, MUC7, MUC13, MUC15, MUC16, MUC18, MUC19, MUC20, MUC21 and MUC22 (reviewed in [11]). Among the mucins in the respiratory tract, there are four gel-forming mucins: MUC2, MUC5AC, MUC5B and MUC19 described so far [12]. Despite the known critical roles of mucins in the respiratory tract, their importance in respiratory viral infections remains largely unknown and further research is warranted.

In this work, we investigated the profile expression of mucins in both human and mouse (MUC/Muc) experimental models, using primary human epithelial cells in vitro as well as in an experimental mouse model of HMPV infection. Based on the observation that mucin 19 was predominantly expressed upon HMPV infection, the role of Muc19 in HMPV-induced pathogenesis was investigated, using a Muc19 deficient mouse model. Our results indicate that Muc19 not only contributes to the HMPV-induced disease and lung viral titers, but also to CD4+T-cell responses. Taken together, these findings suggest that Mucin 19 contributes to HMPV-induced pathogenesis.

## 2. Results

### 2.1. Predominant Expression of MUC19 in Primary Human Cells

**Figure 1:**
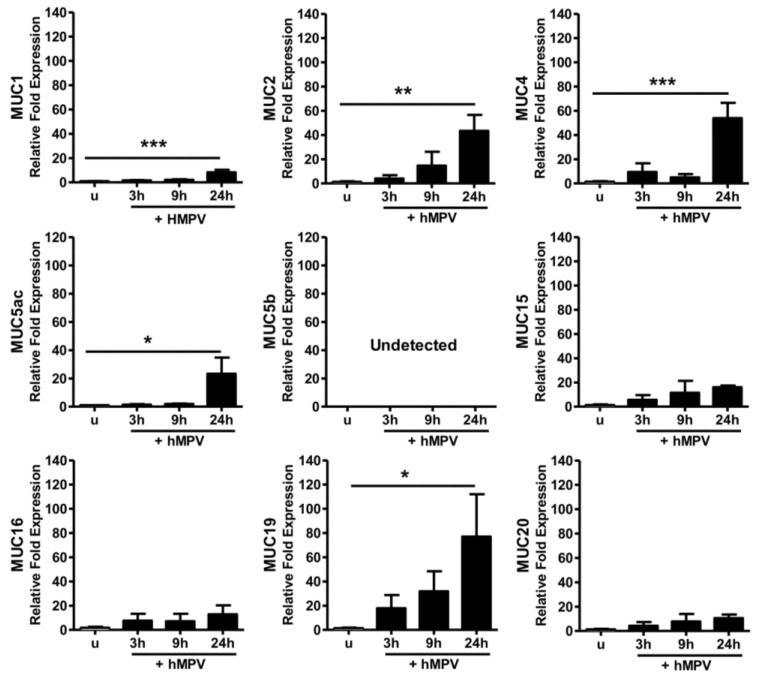
**Mucin expression in normal human bronchial epithelial cells.** Normal human bronchial epithelial (NHBE) cells were infected at an MOI of 3, and RNA was extracted at indicated time points after infection. Mucin expression was evaluated via quantitative real time RT-PCR (qRT-PCR). Graph bars show the average of three independent experiments ± standard error of the mean. One-way analysis of variance (ANOVA) and Dunnett post-test was applied. * *p* < 0.05; ** *p* < 0.01; *** *p* < 0.001.

We initially explored the expression of mucins induced by HMPV in vitro, using normal human bronchial epithelial cells (NHBE). NHBE cells were infected at a multiplicity of infection (MOI) of 3, and mucin expression was analyzed by quantitative real time RT-PCR (qRT-PCR) at 3, 9 and 24 h. As shown in Figure 1, compared to uninfected cells, HMPV infection induced a significant fold-increase expression of MUC1 (8.3 ± 2), MUC2 (43.4 ± 13), MUC4 (54 ± 12), MUC5ac (23.3 ± 11.5) and MUC19 (77 ± 35), while no significant induction of MUC15, MUC16 and MUC20 was observed. MUC5b was not detected. Of note, MUC19 was the most abundantly expressed in response to HMPV infection.

### 2.2. Predominant Expression of Muc19 in the Lungs of Infected Mice

**Figure 2:**
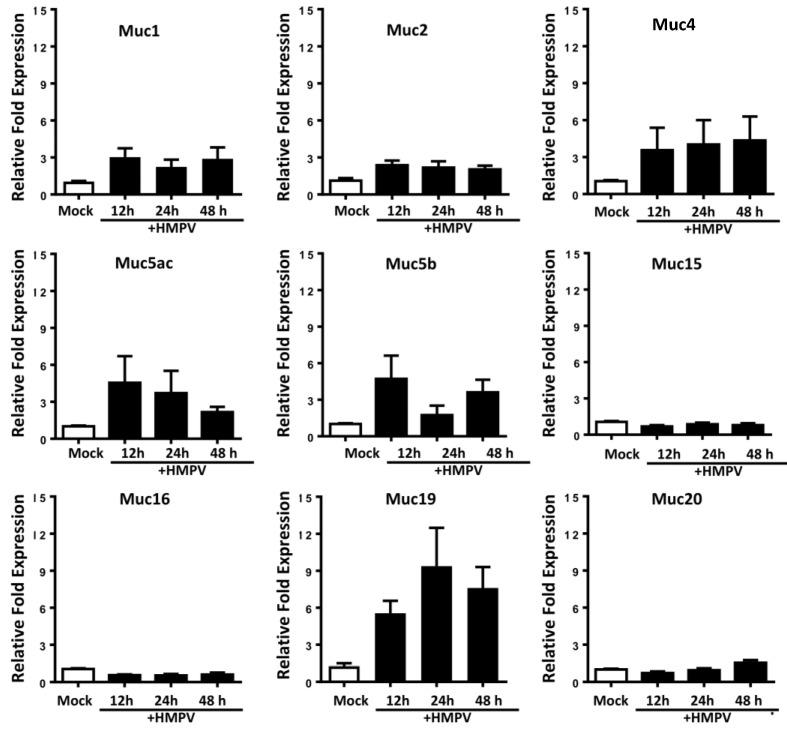
**Mucin expression in the lungs of human metapneumovirus** (**HMPV)-infected mice**. BALB/c mice were infected intranasally with 1 × 10^7^ PFU of HMPV. Lung tissue was obtained at the indicated time points, followed by RNA extraction. Mucin expression analysis was performed by qRT-PCR. Graph bars represent the mean of three independent experiments ± standard error of the mean (n = 4–6). One-way analysis of variance (ANOVA) and Dunnett post-test was applied. No statistical differences were noted.

Next, we characterized the mucin response ex-vivo by analyzing the mRNA expression in the lungs of HMPV-infected mice. BALB/c mice were infected with HMPV, and the expression of Muc1, Muc2, Muc4, Muc5ac, Muc5b, Muc15, Muc16, Muc19, and Muc20 were analyzed at 12, 24 and 48 h after infection. As observed in Figure 2, the expression of Muc15, Muc16 and Muc20 was almost undetectable in HMPV-infected mice. On the other hand, although not statistically significant, HMPV induced a higher expression (1.8 to 4.7 fold) of Muc1, Muc2, Muc4, Muc5ac, and Muc5b when compared to mock-infected mice. Interestingly, we observed that HMPV induced a higher fold increase in Muc19 (5.5 to 9.4 fold) when compared to the other mucins analyzed.

### 2.3. Muc19 Contributes to Viral Replication and to an Optimal CD4+ T-cell Response in the Lung of HMPV-Infected Mice

**Figure 3:**
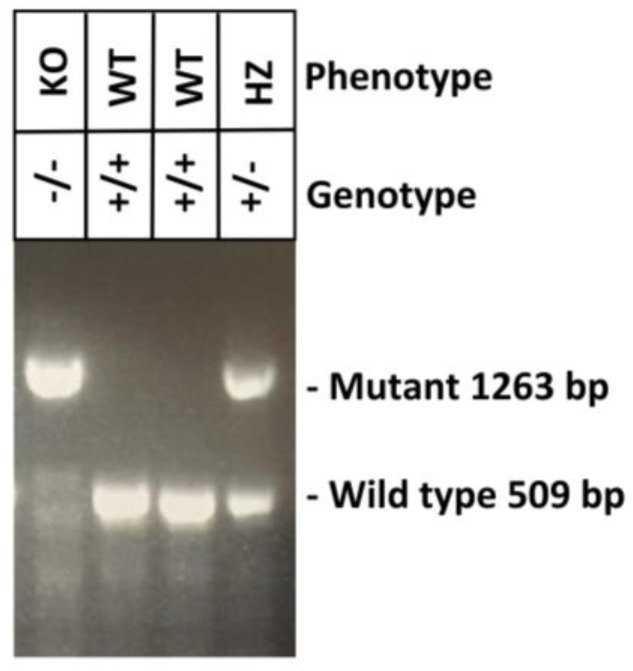
**Muc19 mouse genotyping.** Representative data of PCR-based analysis of genomic DNA from Muc19 mice. Agarose gel electrophoresis shows PCR products from mouse tail samples. The lower band is the wild-type allele of 509 bp, while the upper band corresponds to the mutant allele of 1263 bp. KO, knockout; WT, wild type; HZ, heterozygote.

Based on the data shown in Figure 1 and Figure 2, indicating that HMPV induces a predominant induction of Mucin 19 in human (MUC19) and in mouse (Muc19) models, we further investigated the physiological relevance of Muc19 during HMPV infection. For this work, we used a previously reported Muc19 genetically engineered mutant mice [13]. All mice used in the experiments were genotyped by PCR. Genomic DNA was obtained from tail snips followed by a Touch-down PCR to define the mice genotype. The phenotype of each mouse was then defined according to the genotypes shown in the agarose gel shown in Figure 3. 

**Figure 4:**
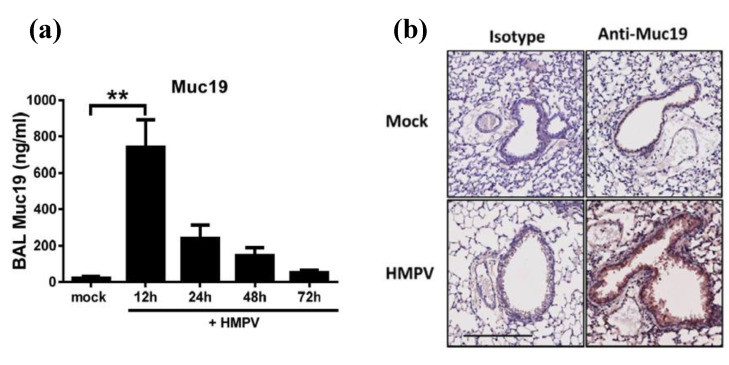
**Muc19 is the predominant mucin expressed in the lungs during HMPV infection.** Muc19^+/+^ mice were infected intranasally with HMPV. Bronchoalveolar lavage (BAL) samples were collected at 12, 24, 48, and 72 h after infection. Expression of Muc19 in the airways was determined by (**a**) ELISA and by (**b**) immunohistochemistry staining in lung tissue sections. Bar graph represent mean ± standard error of the mean (n = 6 mice). Bar = 200 μm. Data are representative of 2 separate experiments with similar results. One-way analysis of variance (ANOVA) and Dunnett post-test was applied. ** *p* < 0.01.

After validation of the Muc19 mouse model, we first quantified the release production of Muc19 in the lungs of infected mice. For that effect, Muc 19^+/+^ (WT) mice were infected with HMPV or were mock infected before bronchoalveolar lavage (BAL) samples were collected at 12, 24, 48, and 72 h after infection. BAL samples were tested for Muc19 protein content by ELISA assay. In addition, lung tissue was fixed and paraffin embedded, followed by immunohistochemistry staining with anti-Muc19 antibody. The data shown in Figure 4a indicate a significant spike in Muc19 as early as 12 h p.i, while levels slowly decreased out to 72 h p.i. Expression of Muc19 was also detected in lung tissue sections at 12 h p.i., as shown in the lower right panel of Figure 4b.

**Figure 5:**
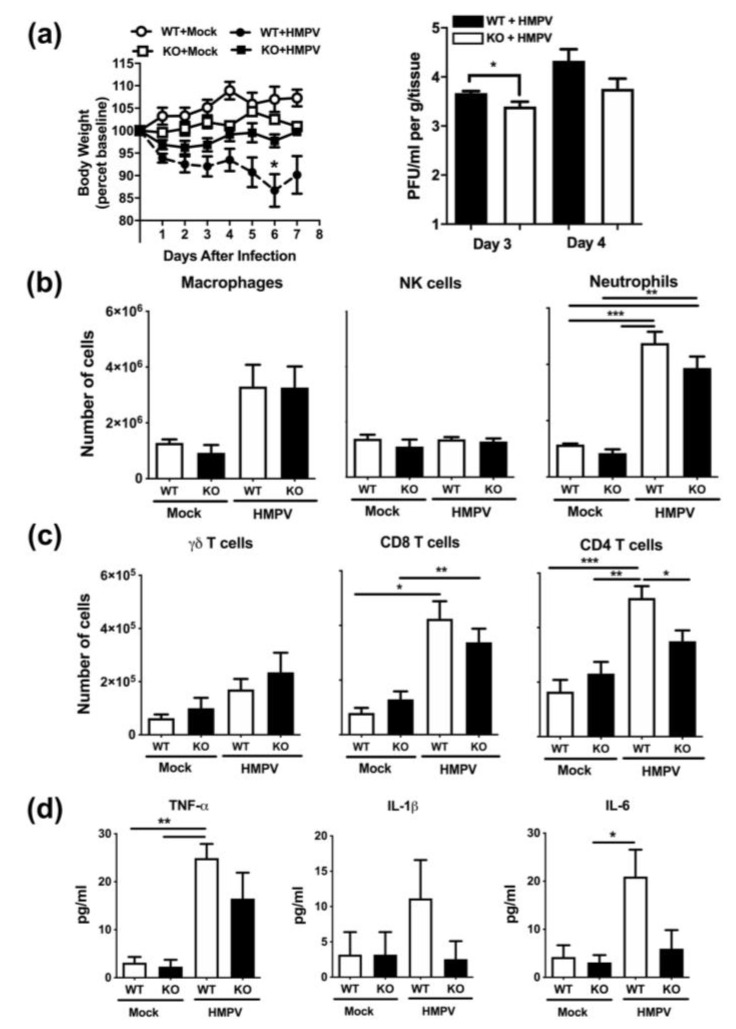
**Muc19 KO mice have altered host response to HMPV infection**. Muc19 WT and Muc19 KO mice were infected intranasally with HMPV or were mock infected. (**a**) Body weight was monitored daily, and lung viral titers were determined by plaque assays at day 3, 4. (**b**) Lung cell recruitment was determined by flow cytometry analysis at day 1 and (**c**) also at day 7. (**d**) Release of pro-inflammatory cytokines was determined in BAL samples by multiplex assays. Bar graph represent mean ± standard error of the mean (n = 7 mice). Data are representative of 3 separate experiments with similar results. One-way analysis of variance (ANOVA) and Tukey post-test was applied. * *p* < 0.05; ** *p* < 0.01; *** *p* < 0.001.

Using the Muc19^-^/^-^ (KO) mice, we then explored the effect of this mucin on HMPV-induced pathogenesis. First, we investigated the effect of Muc19 on body weight loss and viral replication. Mice were infected with HMPV or were mock infected with PBS. Body weight was monitored daily and lung samples were collected at days 3, 4, and 8 after infection. Quantification of infectious viral particles was determined by a methylcellulose plaque assay. As shown in Figure 5a, mice lacking Muc19 lost less weight and recovered their baseline body weight faster than the wild-type ones. Moreover, we observed that the absence of Muc19 led to a modest, yet significant, reduction in lung titers at day 3 after infection. The same trend was observed at day 5. No virions were detected in any mice at day 8 after infection (not shown).

During further investigation of the response to HMPV in the absence of Muc19, we initially analyzed the lung innate cellular infiltration after HMPV infection. Lung tissue was collected at day 1 after infection and digested enzymatically. Lung single cell suspensions were stained with specific combinations of antibodies to identify the frequency of innate immune cells including neutrophils (Ly6G^+^/CD11b^+^), macrophages (F4/80^+^/CD11b^+^) and NK cells (NK1.1^+^). As shown in Figure 5b, there was an increased infiltration of neutrophils and interstitial macrophages upon HMPV infection. However, there were no outstanding differences in cellular content between the Muc19-WT and Muc19-KO animals infected with HMPV.

In order to explore the effect of Muc19 on host immune responses at a later time of infection, lung cellular infiltration was also analyzed at day 7 after infection, when T-cell recruitment is increased in the lungs of HMPV-infected mice [14]. Lung cell suspensions were stained with specific combinations of antibodies to identify CD4+ (CD3e+/CD4+), CD8+ (CD3e+/CD8a+) and γδ (γδTCR^+^/CD3e^+^) T cells. The results of these experiments indicate that there was a significant decrease in CD4+ T cells in the lungs of HMPV-infected muc19 KO mice (Figure 5c). However, that difference did not extend to γδ or CD8+ T cells (Figure 5c).

Based on the observed effect of Muc19 on CD4+T cells, we investigated further changes at the same time point. The release of pro-inflammatory cytokines was determined in BAL samples collected at day 7 after infection, and concentrations of IL-1β, IL-6 and TNF-α were determined by multiplex immunoassay. Data shown in Figure 5d indicate that in the absence of Muc19 there was a trend in decreased production of the pro-inflammatory cytokines, although no significant differences between WT and KO infected mice were found.

## 3. Discussion

Human Metapneumovirus (HMPV) remains one of the most common viral causes of acute respiratory tract infections, especially in children, elderly, and immunocompromised populations. Clinical symptoms can range from mild respiratory disease to severe bronchiolitis and pneumonia, some of which require hospitalization [15,16]. Because the lungs are one of the largest mucosal surfaces in the human body and contain mucus with a vast variety of mucin proteins, further studies are necessary to define the role of these various mucins, both in normal functioning lungs, as well as injured and infected lungs. This work demonstrates that not only is Muc19 expressed in the lungs of infected mice, but it also contributes to HMPV-induced pathogenesis and infection.

The primary functions of mucus include: facilitating the removal of material from the lungs to protect the epithelial surface from injury, playing a role during the pathogenesis of chronic lung diseases involving airway inflammation, and effecting the host’s susceptibility to infection. Mucus is known for its role as a passive barrier, but it also has many important functions in innate defense and regulating epithelial homeostasis [17]. Here, we observed that HMPV induced a predominant expression of MUC19 in primary normal human bronchial epithelial cells (NHBE). We have previously reported that HMPV also induced MUC19 in A549 cell line (human alveolar basal epithelial cells) [18], although no significant induction was observed in those cells. We attribute the MUC19 expression differences to the inherent nature of the cell type and origin. As reported by others, NHBE and A549 can differentially respond to stimuli [19]. In addition, the expression of Muc19 in the respiratory tract upon HMPV infection was confirmed in vivo with the mouse model. When compared to the other gel-forming mucins, MUC19 is highly understudied and this gap in knowledge is even more apparent in regards to MUC19 in the respiratory tract. To date, very limited information is known about this mucin and is only related to the regulation and some potential functional implications. MUC19 has been found in patients with Sjögren syndrome [20] and in middle ear epithelium [21]. Moreover, murine Muc19 has been reported in mouse models of allergy [22] and of mucous cell deficiency within salivary glands [23]. However, to the best of our knowledge, this is the first piece of evidence that demonstrates that Muc19 is induced in the lungs.

While the primary role of mucins is to act as a surface protectant, many have secondary functions within the disease process. Here, we observed that the lack of Muc19 led to a reduced body weight loss and decreased lung viral titers, suggesting that Muc19 contributes to HMPV replication. In line with these observations, mucin glycosylation has been reported as one of the important processes to contribute to IAV replication in epithelial cells [24]. On the other hand, mucins have also shown an antiviral effect as observed in human milk mucins inhibiting rotavirus replication [25]. Likewise, HIV replication is inhibited by several purified mucins from breast milk (MUC1) [26,27]; saliva (MUC5B and MUC7) [28,29,30]; and pregnancy plug mucus (MUC1, MUC2, MUC5AC and MUCB) [31]. Together, these evidences highlight the importance of mucins to inhibit or contribute to viral replication. Hence, further investigation is needed to fully understand mechanisms underlying those effects.

Although mucins are generally known for their role as protective barriers in innate immunity, experimental evidence indicates that mucins can regulate both the number, as well as the type of T-cell response [32,33,34]. One of the novel findings in the current work is the observed reduced frequency of CD4+ T cells in the lungs of Muc19 KO mice infected with HMPV. This observation is important because previous work has shown the contribution of CD4+ T cells in HMPV-induced pathogenesis, where mice depleted of CD4+ T cells with moAb showed an improved disease and less inflammatory response [14]. The correlative observations in this work that Muc19 KO mice had less CD4+ T cells, less proinflammatory cytokines and less weight loss suggest that Muc19 could contribute to the HMPV-induced pathogenesis through a mechanism involving CD4+ T cells. In line with our findings, experimental evidence demonstrates that MUC1 inhibits the proliferation of human CD3+ T cells, which is attributed to its expression on both CD4+ and CD8+ T cells [35,36]. Similarly, additional work has demonstrated that MUC1 is expressed on the majority of T regulatory cells (CD4+/CD25+/Foxp3), which could provide co-stimulatory or co-inhibitory signals depending also on the number of accessory cell ratios present [37]. On the other hand, data in mice deficient of Muc2 demonstrate that the lack of this mucin increases the frequency of Th17 [38]. Overall, this experimental evidence demonstrates that mucins can regulate the adaptive immune response. However, the underlying mechanisms of these effects are not fully understood and warrant further research.

## 4. Materials and Methods

### 4.1. Virus Stocks

HMPV (strain CAN97-83) was grown and titrated in LLC-MK2 cells (ATCC) in the presence of trypsin (Worthington, Lakewood NJ, USA), and virus stocks were generated by centrifugation, as previously described [39].

### 4.2. Cell Lines and Infection in Vitro

Normal Human Bronchial Epithelial (NHBE) cells were purchased from Lonza (Lonza, Houston, TX, USA). NHBE cells were propagated in BEGM™ medium and cells were not used beyond passage 3. Cells were infected with HMPV at an MOI of 3. At the indicated time points, cell lysates were collected and processed for RNA isolation and subsequent analysis.

### 4.3. Mice and Infection Protocol

BALB/c female mice were obtained from Envigo. Muc19^+^/^+^ (WT) and Muc19^−^/^−^ (KO) (NFS/N.Cg-*Muc19*^tm2.1Culp^/Mmucd), were purchased from the Mutant Mouse Resource & Research Centers (MMRRC) at U.C. Davis. Homozygous KO mice displayed no discernable physiological defects. Mice were bred and phenotype was confirmed by genotyping. Both male and female mice were used for these experiments, according to availability. All mice were housed in specific pathogen-free conditions in accordance with the Louisiana State University Institutional Animal Care and Use Committee (protocols 15-055, 18-135, and 17-029). Five- to 8-week-old mice were used in all experiments. Under light anesthesia, mice were infected intranasally (i.n.) with 50 µL of HMPV diluted in PBS at a final administered dose of 1 × 10^7^ PFU [9,40]. As mock treatment, mice were inoculated with an equivalent volume of PBS (herein referred as mock).

### 4.4. Genotyping of NFS/N.Cg-Muc19^tm2.1Culp^/Mmucd Mice

Genomic DNA from each litter was extracted from tail snips using a Qiagen DNeasy kit (Qiagen, Germantown, MD, USA) following the manufacturer’s instructions. Mouse genotyping was done according to the protocol developed by MMRRC at U.C. Davis. Briefly, DNA was subjected to Touch-Down cycling PCR protocol using a mixture of 3 primers (36652-comF, 36652-wtR, 36652-muR) and the following cycling conditions: the first 10 cycles anneal at 65 °C decreasing in temperature by 1 °C; the next 30 cycles anneal at 55 °C. PCR products were run on 1.5% agarose gel for 90 min to identify Muc19 KO (1263 bp band) or WT (509 bp) mice.

### 4.5. Sample Collection

Mice were euthanized by combination of intraperitoneal injection of ketamine and xylazine and exsanguination via the femoral vessels. Bronchoalveolar lavage (BAL) samples were obtained by inserting a cannula into the trachea and flushing 1 mL of PBS into the lungs twice. Cell-free supernatants were kept at −75 °C until further analysis. For lung cell analysis, lungs were perfused and collected on ice until further enzymatic digestion to obtain a single-cell suspension, as previously described [40]. To determine viral load, whole lungs were flash frozen in liquid nitrogen and stored at −75 °C until analysis.

### 4.6. Quantitative Real-Time RT-PCR (qRT-PCR)

Human and mouse samples were processed to extract RNA using an RNeasy Plus Kit (Qiagen, Germantown, MD, USA). Mucins gene expression was determined using specific primers and probes (Integrated DNA technologies, Newark, NJ, USA). Quantification of the gene expression was determined by qRT-PCR, as we previously reported [9,18]. Briefly, qRT-PCRs were run using predesigned assay primers (Integrated DNA Technologies) on a 7900HT a fast real-time PCR system following the manufacturer’s suggested cycling parameters (Applied Biosystems, Waltham, MA, USA). The comparative cycle threshold (ΔΔCT) method was used to quantitate the expression of target genes that was normalized to the endogenous GAPDH expression levels. Fold expression of target genes after HMPV infection was further calculated in reference to normalized transcripts from uninfected cells or mock-infected animals.

### 4.7. ELISA

Levels of murine Mucin 19 (Muc19) were determined in BAL fluid samples by ELISA (Cusabio, Houston, TX, USA), according to manufacturers’ guidelines. The range of the sensitivity of the assay is 1.56 to 100 ng/mL.

### 4.8. Flow Cytometry

Flow cytometry assays were done as we have previously described [40]. Briefly, lung single-cell suspensions were incubated with anti-CD16/32 antibody at 4 °C for 30 min before staining with different combinations of specific antibodies: anti-CD8α, anti-CD3ε, anti-CD4, anti-IA/IE, anti-CD11b, anti-CD161b/161c, and anti-Ly6G, all from BD Pharmingen, and anti-F4/80 from eBioscience. After application with specific antibodies, cells were incubated for an additional 30 min, in the dark, at 4 °C before being washed with PBS/1%BSA before fixing them with 2% paraformaldehyde. Cells were analyzed using a FACScan flow cytometer (BD Biosciences, San Jose, CA, USA) and FlowJo software (v10.3, Tree Star, Ashland, OR, USA). Total numbers of lung cells were counted by trypan blue before flow cytometry analysis. The number of plotted specific cells was calculated based on the percentage obtained by flow cytometry analysis and the total number of lung cells.

### 4.9. Immunohistochemistry

Detection of Muc19 in the lungs of infected mice was done in a multi-step staining process. First, antigen retrieval was performed by adding 200 mL 1x citrate buffer solution (Dako, Target Retrieval Solution, Ref S1699, Santa Clara, CA, USA) to Coplin jar. Then the basin of decloaker (Biocare Medical, DC2002, Pacheco, CA, USA) was filled with 500 mL of DH_2_O, while the Coplin jar was placed in the center. Temperatures were: SP1−125 °C for 30 s, and SP2−90 °C for 10 s. Slides were then cooled for 10 min and rinsed three times with dH_2_O. The whole process was then repeated again, but the Coplin jar was filled with 200 mL of 2x tris-buffered saline and tween (TwBS) (Thermo Scientific, Ref TA-999-TT, Waltham, MA, USA).

After antigen retrieval, endogenous enzymes were blocked with 3% hydrogen peroxide (Vi-Jon, L0011379BA) for 10 min. Then, the sample was incubated for 30 min with normal rabbit serum (Vector Laboratories, S-5000) diluted 1:50 in TwBS. Anti-Muc19 antibody (Abcam, ab121014, Cambridge, MA, USA) was diluted 1:250 with the Dako Antibody diluent (Dako, Ref S0809, Dako-Agilent, Santa Clara, CA, USA) and incubated for 30 min. Secondary antibody was a biotinylated anti-goat IgG (Vector Laboratories, BA-5000), diluted 1:200 in TwBS, and incubated for 30 min. For Ag detection, a slide was incubated for 30 min with an avidin/biotin complex (“Vectastain ABC-HRP Kit”, PK4000, Vector Laboratories, Burlingame, CA, USA). The chromogen used was impact Nova Red, Peroxidase substrate kit (SK-4805, Vector Laboratories, Burlingame, CA, USA), incubated for 8 min, followed by a 5 min incubation of hematoxylin (Ref 812, ANATECH LTD, Battle Creek, MI, USA) for counterstaining. The tris-buffered saline and tween buffer (TwBS) was used as a rinse between each step.

### 4.10. Cytokine Release

The concentration of proinflammatory cytokines (IL-6, IL-1β and TNF-α) in BAL fluid was determined with a Milliplex MAP mouse cytokine detection system (Millipore, Burlington, MA, USA), following the manufacturer’s protocol. The sensitivity range of the assay is 3.2 to 10,000 pg/mL.

### 4.11. Plaque Assay

Lung viral titers were determined by plaque assays as we previously reported. Briefly, whole lungs were homogenized and 2-fold serial dilutions were performed with the supernatant, which was then plated onto LLC-MK2 cells with a methylcellulose overlay. Six days later, plaques were visualized with horseradish peroxidase (HRP) staining as previously described [9].

### 4.12. Statistical Analysis

A one-way ANOVA statistic was used to compare 3 or more sets of values, followed by a Tukey–Kramer test to correct for multiple comparisons. Dunnett’s multiple comparison post-test was applied between experimental groups against the control group. All statistics listed are reported as mean ± SEM. Statistical analyses were performed using Graph Pad InStat 3 (GraphPad Software). *p* < 0.05 was considered statistically significant.

## 5. Conclusions

In conclusion, our studies demonstrate that HMPV induces Mucin 19, which contributes to disease, viral replication and CD4+ T-cell responses in the lungs of HMPV-infected mice. The results shown in this work suggest that Muc19 plays a role in the HMPV-induced pathogenesis through mechanisms that need future investigation. Defining the importance of mucins in the immune response against pathogens is critical to better understand the factors required for regulating the immune response to HMPV and other viral infections in the respiratory tract as well as to provide potential targets of clinical relevance.
